# Smoking status and attitudes toward tobacco control among students in a vocational school in Lanzhou, China: A cross-sectional study

**DOI:** 10.18332/tpc/236281

**Published:** 2026-11-03

**Authors:** Lei Sun, Jiayi Zhao, Huayu Zhang, Lin Wang

**Affiliations:** 1 School of Applied Engineering, Gansu Finance and Trade Professional CollegeLanzhouChina; 2 School of Accounting and Finance, Gansu Finance and Trade Professional CollegeLanzhouChina; 3 Institute of Education, University of ReadingReadingUnited Kingdom; 4 School of Public Health, Lanzhou UniversityLanzhouChina; 5 Graduate School of Horticulture, Chiba UniversityChibaJapan

**Keywords:** vocational college students, adolescents, smoking behavior, nicotine dependence, tobacco control policy

## Abstract

**Introduction:**

Tobacco use remains one of the leading preventable causes of premature death worldwide, and adolescents who initiate smoking early are at a heightened risk of persistent nicotine dependence and long-term tobacco-related morbidity. Smoking behavior among vocational college students, many of whom enter an integrated secondary and higher vocational education system during a high-risk developmental window, is therefore particularly concerning. The aim of this study was to estimate the prevalence of current smoking, to characterize nicotine dependence, and to assess attitudes toward tobacco-control policies among students at a vocational college in Lanzhou, Gansu Province.

**Methods:**

A cross-sectional study was conducted at a single vocational school in Lanzhou, Gansu Province, China. All eligible first- and second-year students who volunteered to participate and provided written informed consent were invited to complete a self-administered, self-reported questionnaire between June and July 2025. Descriptive statistics and chi-squared tests were used to examine associations between exposure variables (sociodemographic characteristics and tobacco-control-related attitudes) and the outcome variable (smoking behavior).

**Results:**

Of the 1683 questionnaires distributed, 1365 valid responses from 654 male and 711 female students were retained (effective response rate 81.11%). Descriptive statistics and chi-squared tests were used to compare smoking behavior and tobacco-control attitudes across student subgroups. The overall smoking rate among students was 11.21%, with males (22.63%) significantly more likely to smoke than females (0.70%) (p<0.05). Using female students as the reference category, being male was associated with higher odds of current smoking (OR=41.30; 95% CI: 16.82–101.43). Students with urban residence reported a higher smoking rate (15.76%) than those from rural areas (10.50%), and current smoking increased with monthly disposable allowance (7.19%, 11.99%, and 28.16% for <1000, 1000–2000, and >2000 RMB, respectively). Most smokers had mild nicotine dependence (75.16%), and smoking most often occurred at home (57.52%) and in dormitories (22.22%). A total of 1142 students (83.66%) supported the implementation of campus tobacco-control policies, and 1074 (78.68%) were willing to participate in school-based tobacco-control education. However, only 64.98% considered the current school measures effective, and 142 students (10.40%) considered smoking in public places acceptable.

**Conclusions:**

The smoking prevalence among students at this Lanzhou vocational school is relatively high and is strongly concentrated among male students. Given the cross-sectional design, causal inferences and the effectiveness of specific interventions cannot be established. The findings nevertheless identify priority groups and attitudes that merit further evaluation in longitudinal and intervention research.

## Introduction

Tobacco use is one of the leading preventable risk factors for death and disease worldwide^1-5^. The smoking prevalence of 21.20% among male college students^6^ was markedly higher than the overall student smoking prevalence of 7.8% from that national surveillance report. Notably, the smoking rate among students in higher vocational colleges (11.6%) was significantly higher than that among students in provincial or municipal universities (5.0%) and those in national or jointly administered institutions (3.0%)^7^. This finding indicates that smoking behavior among vocational college students is particularly concerning, and that youth smoking has become a pressing public health challenge in China^7^. Therefore, regular surveillance of tobacco-use behaviors and evaluation of attitudes toward tobacco control policies among adolescents are critical for youth tobacco-control research^8-11^.

Although many research studies regarding adult smoking have been conducted^1-3,8^, studies on the prevalence of adolescent smoking and associated factors, especially in public full-time higher vocational colleges that implemented an integrated secondary and higher vocational education system, are still lacking. Students in such institutions are mostly aged 16–19 years, a developmental window in which smoking initiation peaks, and they experience closer peer interaction and a stronger sense of social identity, and several majors (e.g. engineering) provide more open practical scenarios in which campus supervision of tobacco use is more difficult than in ordinary secondary schools. These age-related, peer-driven, and supervision-related features make this type of institution a distinctive and high-priority setting for adolescent tobacco surveillance. Hence, a cross-sectional questionnaire survey from June to July 2025 among first-and second-year students at a secondary vocational school was conducted in Lanzhou, Gansu Province.

The specific objectives were to: 1) estimate the prevalence of current smoking among first- and second-year vocational students; 2) identify factors associated with smoking, including sex, household registration, monthly disposable allowance, and self-rated health; 3) assess attitudes toward campus tobacco-control policies among both smokers and non-smokers; and 4) compare these attitudes between smokers and non-smokers. The subgroup comparisons of smoking behavior across demographic categories and of attitudes between smokers and non-smokers were prespecified in the study protocol before data analysis.

## Methods

### Participants

A cross-sectional study was conducted at a vocational school in Lanzhou, Gansu Province, China. All enrolled first- and second-year students were eligible. Exclusion criteria were: 1) students who did not provide consent or declined to participate; 2) third-year or other grades not part of the recruitment frame; and 3) questionnaires with one or more essential items left blank or with logically inconsistent responses (e.g. reporting current smoking while denying ever having tried a cigarette). A total of 1683 questionnaires were distributed, and after excluding those with missing data, 1365 valid questionnaires were obtained, yielding an effective response rate of 81.11%.

### Survey instrument and procedure

The questionnaire was developed and refined based on the 2021 Chinese Youth Tobacco Survey Questionnaire, the Nicotine Dependence Evaluation Scale^12^, and related research^13-16^ (Supplementary file). Data collection was performed using a self-administered, self-reported electronic questionnaire. To ensure data security and confidentiality, the study utilized the Chinese data-sharing platform of the Research Electronic Data Capture (REDCap) system for questionnaire distribution, data collection, and management. With the cooperation of the school administration, all participants completed the electronic questionnaire anonymously. Reliability and validity testing conducted using SPSS version 21.0 yielded a Cronbach’s α coefficient of 0.785, indicating good internal consistency and validity of the instrument.

### Measures and variable definitions

Current smoking was defined as having smoked cigarettes on at least 1 day in the past 30 days and was treated as the primary outcome for prevalence and associated-factor analyses. Nicotine dependence (assessed with the Chinese Fagerström-based test for nicotine dependence scale) and the attitudes toward campus tobacco-control policies were treated as secondary outcomes. Exposure/independent variables comprised sex (male, female), grade (first, second), residence (urban, rural), and monthly disposable living allowance. The allowance was categorized *a priori* as <1000, 1000–2000, and >2000 RMB, with the <1000 category serving as the reference group. These cut-points were chosen to reflect the typical monthly living allowance of vocational students in this region. Sex, residence, and income were treated as potential confounders in interpreting the association between sociodemographic characteristics and smoking. To reduce reporting bias, questionnaires were completed anonymously via the REDCap platform, participants were assured of confidentiality, and neither teachers nor school administrators were present during completion.

### Statistical analysis

Data were cleaned using Microsoft Excel 2021 and analyzed using SPSS version 21.0. Descriptive statistics were expressed as frequencies (n) and percentages (%). Chi-squared tests were used to compare smoking behavior and attitudes across subgroups. Because this was an exploratory, cross-sectional surveillance study, no multivariable logistic regression model was fitted; all reported comparisons used unadjusted (crude) odds ratios (ORs) and were based on chi-squared tests. No subgroup/interaction or sensitivity analyses were performed beyond the prespecified two-group (smoker, non-smoker) and demographic comparisons. Missing data were handled by complete-case analysis; questionnaires with any essential item missing were excluded before analysis, and the 1365 retained questionnaires were complete on all variables used. All statistical tests were two-tailed, and p<0.05 was considered statistically significant.

## Results

### Sample characteristics

A total of 1683 questionnaires were returned from first- and second-year students at the vocational school. Of these, 1365 responses contained no missing values for key study variables and were classified as valid, yielding an effective response rate of 81.11%. Among the respondents, 654 were male (47.91%) and 711 were female (52.09%). The proportions of first- and second-year students were roughly equal, accounting for 46.89% and 53.11%, respectively. A total of 1181 students (86.52%) came from rural areas. Most students reported a monthly disposable living allowance of <1000 RMB (41.76%) or 1000–2000 RMB (50.70%). Regarding self-rated health status, 75.38% of students rated their health as ‘Fairly good’, while 3.15% rated it as ‘Relatively poor’.

### Tobacco use

Among the 1365 vocational school students surveyed, 153 reported current smoking, indicating a smoking prevalence of 11.21%. The smoking rate among males (22.63%) was higher than that among females (0.70%). Students with urban residence had a higher smoking rate (15.76%) than those from rural areas (10.50%). The smoking rates among students with monthly disposable living allowances of <1000, 1000–2000, and >2000 RMB were 7.19%, 11.99%, and 28.16%, respectively. These differences were statistically significant (χ^2^=164.564, χ^2^=4.428, χ^2^=39.387; p<0.05).

The smoking rates of first-year and second-year students were 12.50% and 10.07%, respectively, with no statistically significant difference (χ^2^=2.019, p=0.155). Regarding self-rated health status, students who perceived their health as ‘poor’ had the highest smoking rate (30.77%), followed by those rating their health as ‘average’ (10.58%), ‘fairly good’ (11.30%), ‘good’ (11.45%), and ‘relatively poor’ (3.33%), with no statistically significant differences (χ^2^=7.017, p=0.135) (Table 1).

**Table 1:** Differences in smoking behavior by participant characteristics among vocational school students in Lanzhou, Gansu Province, China, June–July 2025 (N=1365)

Variables	Current smokers n (%)	Non-smokers n (%)	χ²	p	OR	95% CI
**Gender**			164.564	<0.001		
Male	148 (22.63)	506 (77.37)			41.3	16.8–101.5
Female (ref.)	5 (0.70)	706 (99.30)			1	
**Grade year**			2.019	0.155		
First	80 (12.50)	560 (87.50)			1.28	0.91–1.80
Second (ref.)	73 (10.07)	652 (89.93)			1	
**Residence**			4.428	0.035		
Urban	29 (15.76)	155 (84.24)			1.60	1.03–2.47
Rural (ref.)	124 (10.50)	1057(89.50)			1	
**Monthly living allowance** (RMB)			39.387	<0.001		
<1000 (ref.)	41 (7.19)	529 (92.81)			1	
1000–2000	83 (11.99)	609 (88.01)			1.76	1.18–2.62
>2000	29 (28.16)	74 (71.84)			5.06	3.02–8.48
**Self-rated health status**			0.151	0.927		
Fairly good (ref.)	117 (11.37)	912 (88.63)			1	
Average	31 (10.58)	262 (89.42)			0.92	0.62–1.38
Relatively poor	5 (11.63)	38 (88.37)			1.03	0.40–2.64

RMB: Chinese Renminbi. Variables marked (ref.) indicate the baseline comparison group for crude odds‑ratio calculations. Statistically significant at p<0.05.

### Tobacco dependence among smoking students

Among the 153 students who reported smoking, most (75.16%) had mild nicotine dependence, while 18.30% and 6.54% were classified as having moderate and severe dependence, respectively. Regarding smoking locations, the majority of students reported smoking at home (57.52%), followed by in school dormitories (22.22%) and school restrooms (13.73%). A smaller proportion smoked on the school playground (1.31%) or in other open areas on campus (5.23%) (Table 2).

**Table 2:** Nicotine dependence and smoking locations among smoking students in a vocational school in Lanzhou, Gansu Province, China, June–July 2025 (N=153)

Variables	n	%
**Level of nicotine dependence**		
Mild	115	75.16
Moderate	28	18.30
Severe	10	6.54
**Usual smoking location**		
At home	88	57.52
School dormitory	34	22.22
School playground	2	1.31
Outdoor areas on campus	8	5.23
School restroom	21	13.73

### Attitudes toward tobacco control among vocational school students

Among the 1365 vocational school students surveyed, 1107 (81.10%) were aware of their school’s tobacco control policies, with comparable awareness between smokers (84.97%, 130/153) and non-smokers (80.61%, 977/1212); this difference was not statistically significant (χ²=1.682, p=0.195). A total of 1142 students (83.66%) believed that schools should implement tobacco control policies, and this view was significantly more common among non-smokers (84.90%, 1029/1212) than among smokers (73.86%, 113/153) (χ²=12.125, p<0.001). Most students (64.98%) considered the school’s current tobacco control measures effective, with no statistically significant difference between smokers (69.93%, 107/153) and non-smokers (64.36%, 780/1212) (χ²=1.858, p=0.173). Willingness to participate in school-based tobacco control education was expressed by 78.68% of students, and was significantly higher among non-smokers (79.70%, 966/1212) than among smokers (70.59%, 108/153) (χ²=6.728, p=0.009). Only 10.40% of students believed that smoking in public places is acceptable, a perception significantly more common among smokers (24.84%, 38/153) than among non-smokers (8.58%, 104/1212) (χ²=38.515, p<0.001). When exposed to secondhand smoke, most students reported that they would ‘walk away immediately’ (51.21%, 699/1365), followed by ‘persuade the smoker to stop’ (29.60%) and ‘ignore it’ (19.19%). The proportion choosing to walk away was significantly higher among non-smokers (52.48%, 636/1212) than among smokers (41.18%, 63/153) (χ²=14.038, p<0.001) (Table 3).

**Table 3:** Attitudes toward tobacco control by smoking status among vocational school students in Lanzhou, Gansu Province, China, June–July 2025 (N=1365)

Variables	Current smokers n (%)	Non-smokers n (%)	χ²	p	OR	95% CI
**Aware of school tobacco control policy**			1.682	0.195		
Yes	130 (11.74)	977 (88.26)			1.36	0.85–2.18
No (ref.)	23 (8.91)	235 (91.09)			1	
**Believe school should implement tobacco control policy**			12.125	<0.001		
Yes	113 (9.89)	1029(90.11)			0.50	0.33–0.76
No (ref.)	40 (17.94）	183 (82.06）			1	
**Perception of effectiveness of current school tobacco control policy**			1.858	0.173		
Yes	107 (12.06)	780 (87.94)			1.29	0.89–1.87
No (ref.)	46 (9.62)	432 (90.38)			1	
**Willingness to participate in school tobacco control health education**			6.728	0.009		
Yes	108 (10.06)	966 (89.94)			0.61	0.41–0.90
No (ref.)	45 (15.46)	246 (84.54)			1	
**Consider smoking in public places normal**			38.515	<0.001		
Yes	38 (26.76)	104 (73.24)			3.52	2.28–5.44
No (ref.)	115 (9.40)	1108(90.60)			1	
**If you are a passive smoker, what would you do?**			14.038	<0.001		
Walk away immediately (ref.)	63 (9.01)	636 (90.99)			1	
Ignore	46 (17.56)	216 (82.44)			2.15	1.41–3.28
Persuade the smoker to stop	44 (10.89)	360 (89.11)			1.23	0.81–1.86

Variables marked (ref.) indicate the baseline comparison group for crude odds‑ratio calculations. Statistically significant at p<0.05.

## Discussion

This cross-sectional study found that current smoking was concentrated among male vocational students in this Lanzhou vocational school. Although the majority of students supported campus tobacco-control policies and recognized their importance, smokers consistently held less favorable attitudes and perceived fewer risks than non-smokers. These patterns point to the students most in need of tobacco-control attention and to the attitudes that warrant further investigation.

In this study, the current smoking rate among students in a vocational college in Lanzhou, Gansu Province, was 11.21%. Compared with the adolescent smoking rate of 4.7% in 2019 GYTS (China)^17^, the prevalence of tobacco among students in the vocational school was relatively high. As indicated by Littlecott et al.^18^, the smoking rate in vocational schools located in high-income nations is in the range of 8–12%, and even in low socioeconomic status (SES) regions, this indicator generally falls within 15–20%, with our findings nearly reaching the upper boundary of this interval. However, the adolescent smoking rate in our research was similar to that of domestic vocational schools in Shanghai (11.23%)^19^. Notably, the overall smoking rate of adolescents in Shenzhen was 2.3%, which might be due to the revision of tobacco control regulations, known as one of the most stringent tobacco control regulations in China, by the Shenzhen government in 2019^20^. Developing policies for adolescent tobacco control was essential, while campus tobacco control management remains insufficient in vocational colleges.

Additionally, the smoking rate among male students was 22.63% higher than that of female students (0.7%); a similar pattern has also been reported, where male students (9.6%) outperform female students (1.9%)^17^. The relatively higher smoking rate of male students in higher vocational colleges was also reported in the 2021 China College Student Tobacco Use Survey (21.20%)^6^. Males are more susceptible to direct coercion, whereas females are more likely to be influenced by subtle encouragement^17,18^. These findings suggest that differences in student composition, institutional management, and campus environments may contribute to the disparities between vocational and general universities. Furthermore, male students remain a key target population for tobacco control interventions, consistent with previous research findings^21-26^.

In our study, current smoking was associated with distinct attitudes toward tobacco control. Compared with non-smokers, a smaller proportion of smokers regarded smoking in public places as unacceptable (75.16%, 115/153 vs 91.42%, 1108/1212), supported campus tobacco control policies (73.86%, 113/153 vs 84.90%, 1029/1212), or expressed willingness to participate in tobacco control education (70.59%, 108/153 vs 79.70%, 966/1212). These group differences were consistent with the theory of Ferrer et al.^27^ that health-related harm perceptions are fundamental to many health behavior changes. Misconceptions about tobacco were significantly and positively associated with the risk of smoking behavior among adolescents^28,29^. This association is common across substances and contexts. A previous study related to misconceptions about cannabis secondhand smoke (cSHS) reported that US adults who perceived cSHS as ‘extremely harmful’ were 6.0 times more likely to implement a complete ban on in-home cannabis smoking (OR=6.0; 95% CI: 4.9–7.2) compared to those who considered it ‘totally safe.’ A significant dose-response relationship was notably observed in this study: the higher the perceived harm, the greater the likelihood of adopting smoke-free rules^29^. This phenomenon might be attributed to the transmission mechanism on health risk perception-behavioral attitude: low perceived harm weakens individuals’ recognition of tobacco control rules^27,29^. For instance, in a previous study, 28.6% of participants who perceived cSHS as ‘safe’ had a household smoke-free rate of only 36.8–53.9%^29^. Similarly, in this study, students who considered ‘smoking in public places acceptable’ (mostly smokers) showed <10% support for tobacco control policies, reflecting the inhibitory effect of cognitive biases on tobacco control participation.

In addition, adolescents who perceived smoking as more attractive and comfortable in social environments, peers' smoking behavior contributed to initiating and maintaining cigarette use among adolescents^30^. The residual effect of smoking as a ‘social tool’ is particularly prominent in vocational schools. In this study, smoking students exhibited a higher rate of incorrect perceptions regarding public smoking, suggesting that some students still view smoking as an ‘acceptable social behavior’ – consistent with findings from Dutch vocational school studies where ‘smoking students gather in groups during breaks to strengthen a sense of belonging’^31,32^. A previous international multi-context study confirmed that peer influence was a critical factor in adolescent smoking, regardless of whether smoking was ‘normalized’ or ‘denormalized’. During the normalization period, smoking served as a tool for group belonging; in the denormalization era, smoking groups shifted to covert socialization, but peer pressure remained^18^. This conclusion is indirectly supported by our study: the low support for tobacco control among smoking students (9.89%) might be related to peer smoking. Their avoidance of tobacco control information also reflects the high tolerance for smoking within their social circles.

Compared to international vocational school students and domestic general adolescents, the vocational school students in this study exhibit significant uniqueness in smoking rates, policy perceptions, and behavioral responses. These differences are the core targets for precise tobacco control. Developing effective tobacco control policies among adolescents was the core driver. A study shows that adolescent smoking behaviors became more covert, negative attitudes toward smoking increased, and school tobacco control policies indirectly inhibited smoking by reinforcing the norm of ‘smoking not being allowed’^18^. Domestic studies in Shenzhen have also confirmed that the adolescent smoking rate (2.3%) was significantly lower than the national average (5.9%) after the implementation of strict tobacco control regulations^20^. In our study, 81.10% of vocational school students were aware of campus tobacco control policies, 91.69% expressed support, and 64.98% recognized their effectiveness, demonstrating characteristics of ‘high awareness and high support’.

However, it is important to note that the effectiveness of policies varies across groups. Overall policy awareness was high (81.10%); although smokers showed slightly lower within-group awareness (84.97% vs 80.61%), this difference was not statistically significant (p=0.195), suggesting that awareness is broadly comparable across groups. Moreover, in our study, 51.39% of students chose to ‘leave immediately’, and 35.90% opted to ‘discourage smoking’; the core characteristic remains avoidance first. Further analysis revealed that non-smoking students were significantly more likely to ‘leave immediately’ than smoking students (p<0.001), reflecting differences in tolerance for secondhand smoke between the two groups. This individual protection-oriented response could reduce personal exposure but failed to improve the overall tobacco control environment, creating a gap with the international trend of active intervention to build a smoke-free atmosphere^18^.

### Strengths and limitations

The most significant strength of this study lies in its analysis of the specific group of adolescents, characterizing their tobacco use and associated factors, which will contribute to providing critical evidence to inform the development of tailored, evidence-based tobacco control interventions by government and public health authorities.

However, the present study also has some limitations. While some variables such as socioeconomic status (SES), e-cigarette use, and differences across majors were emphasized in international literature, these factors were not analyzed in our study. The lack of gradient cognitive questions (e.g. ‘totally safe to extremely harmful’) made it impossible to verify the dose-response relationship. The cross-sectional study design provides no inferences about causality. In addition, the current smoking status was self-reported, and no biological validation (e.g. cotinine measurement) was applied. Self-reported smoking can introduce information bias and misclassification of the outcome; participants tended to under-report smoking owing to socio-cultural norms, which may attenuate or distort the observed associations^30^. Qualitative interviews to support the exploration of smoking motivations were also lacking. Because no multivariable adjustment was performed anywhere in the analysis, residual confounding by measured (e.g. sex, income) and unmeasured factors cannot be ruled out, and all reported associations should be interpreted as unadjusted. Also, this study was conducted in a single vocational school in Lanzhou over a two-month period; the findings may not be generalizable to vocational schools in other regions, to other institution types, or to students in later grades; replication in larger, multi-site samples is warranted. Despite limitations, our study will help lay a foundation for future research on tailored anti-tobacco policies and measures for adolescents, and further longitudinal research is still needed.

### Implications

This study yields valuable insight into the associated factors of adolescent smoking behavior in vocational college, with implications for targeted tobacco control interventions. Vocational school students are mostly aged 16–19 years and are in a high-risk period for smoking attempts. In addition, some majors (such as engineering) have more open practical scenarios, and the difficulty of tobacco control supervision is greater than that of ordinary middle schools. Future tobacco control policies should be designed with more targeted interventions and stricter intervention measures (including disciplinary action and establishing a smoking ban supervision post) for the smoking population. Teenagers are in a critical period of social identity. Vocational school students have closer peer interactions, and if people around them smoke, it can reduce their perception of the harms of smoking while increasing opportunities to access tobacco. Contributions of parents, teachers, and peers to smoking should be more focused on developing school-based cessation, linking multiple scenario regulations with family interventions to build a collaborative system: strengthening tobacco control in outdoor training scenarios, establishing student supervisors, and coordinating monitoring with professional teachers could be effective and tailored measures. Meanwhile, future research could be improved by supplementing data on SES, majors, and family smoking status to verify the Littlecott et al^18^. ‘SES gradient effect’, as well as the Tripathi et al^29^. ‘family moderating effect’. Moreover, future studies could adopt a mixed-methods design, combining quantitative surveys with in-depth interviews of smoking students to explore the underlying psychology of their ‘active avoidance of policies’. Besides, intervention studies could be conducted in the future to verify the effectiveness of the ‘non-critical counseling + scenario-based supervision’ model, providing empirical support for tobacco control in vocational schools.

## Conclusions

This study found a relatively high smoking prevalence among vocational-school students in Lanzhou, particularly among males, together with overall strong support for and awareness of campus tobacco-control policies but lower acceptance of control measures among smokers. Within-group policy awareness was comparable between smokers and non-smokers, whereas attitudes toward public-smoking acceptability and control-policy support differed markedly between the two groups. Given the cross-sectional design, the effectiveness of interventions cannot be inferred; the priority groups and attitude gaps identified here provide a basis for future longitudinal and intervention studies tailored to the vocational-education setting.
