# Supplementary material for: Smoking status and attitudes toward tobacco control among students in a vocational school in Lanzhou, China: A cross-sectional study
Source: Tob Prev Cessat. 2026 Nov 3;12:10.18332/tpc/236281. doi: 10.18332/tpc/236281 (PMC13736817; doi:10.18332/tpc/236281)
Supplement: Supplementary file 1 [file TPC-12-68-s001.pdf]

TOBACCO PREVENTION AND CESSATION

Supplementary file

© 2026 Sun L. et al.

DOI:
10.18332/tpc/236281

The content has been provided by the author(s) and has not been reviewed, verified, or endorsed by European Publishing. It may not have undergone peer review. The views, opinions, and recommendations expressed are solely those of the author(s) and do not necessarily reflect the position of European Publishing. European Publishing accepts no responsibility or liability for any consequences arising from the use of, or reliance on, this content.

青少年控烟与健康行为、态度调查问卷

亲爱的同学:

你好！我们是兰州大学公共卫生学院青少年控烟与健康行为、态度调查工作组。本调研旨在了解青少年对于控烟的健康行为与态度，为校园控烟政策的完善与实施提供依据。

调研采取匿名作答，你的回答无所谓对错，所收集的数据仅用于本研究，任何渠道下均不会公布个人信息。如果你同意参加，请在下面方框打勾并点击下一步，开始答题。

青少年控烟与健康行为、态度调查工作组

2025 年 7 月

感谢你的参与！请仔细阅读并回答以下问题，除特殊注明外，大部分题目为单项选择，请选择最符合您的情况的选项，开放式问题请在问答框内作答。

基本情况 Basic Information

1. 你所在的年级：What is your grade?

A：2023 级 Grade 2023

B：2024 级 Grade 2024

2. 你的专业？

A：工程造价

B：食品检验检测技术

C：智能建造技术

D：建设工程管理

E：粮食工程技术与管理

F：粮食储运与质量安全

G：建筑经济信息化管理

3. 你的性别？

A：男

B：女

4. 你的年龄为_____岁？

5. 你的户籍所在地？

A：城市

B：农村

6. 你每月的生活费大约为_____元？（不包括学费）

7. 你认为自己的健康状况？

A：好

B：比较好

C：一般

D：比较差

E：差

烟草使用情况

8. 本学期开学前你是否吸烟？

A：是

B：否

9. （设置跳转，仅第 8 题选择“是”的需要作答）此前你大约每天吸多少支烟？_____

10. 尼古丁依赖程度评价（Fagerstrom Test for Nicotine Dependence, FTND）（下面是一些关于日常生活的描述，请根据真实情况，选择最符合您最近情况的一项，并在相应的方格内打“√”。）

评估内容	0 分	1 分	2 分	3 分
a.您早晨醒来后多长时间吸第一支烟？	60 分钟后	31-60 分钟内	6-30 分钟	5 分钟内
b.您是否在需要禁烟场所很难控制吸烟的需求？	否	是		
c.您认为哪一支烟您最不愿意放弃	其他	早晨第一支		
d.您每天吸多少烟？	10 支及以下	11-20 支	21-30 支	31 支及以上
e.您卧病在床时仍旧吸烟吗？	否	是		
f.您早晨醒来后第一个小时是否比其他时间吸烟多？	否	是		

11. 你通常在哪里吸烟?

A: 家里

B: 学校宿舍

C: 学校操场

D: 教学楼

E: 校园露天场所

F: 学校厕所

C: 其他场所（请填写具体场所: _____）

能力因素

12. 你认为吸烟可能导致以下哪些健康问题？*(多选)

A: 中风

B: 骨质疏松

C: 肺癌

D: 心脏病发作

E: 牙齿发黄

F: 男性精子质量减少

G: 大脑发育损伤

H: 弱视

13. 你认为吸入二手烟（被动吸烟，即非自愿吸取其他吸烟者喷吐的烟雾的行为）可能导致以下哪些健康问题？*（多选）

A: 肺癌

B: 呼吸道感染

C: 中风

D: 心脏病

E：支气管炎

F：哮喘

G：中耳炎

14. 你知道电子烟吗？

A：是

B：否

15. 你是否吸过电子烟？

A：是

B：否

16. 你认为电子烟对人体是否有害？

A：是

B：否

17. 自我效能评价（下面是一些关于吸烟自我认知的描述，请根据真实情况，选择最符合您最近情况的一项，并在相应的方格内打“√”。）

评估内容	肯定不会	可能不会	可能会	肯定会
a. 如果有好朋友给你香烟，你会吸吗？				
b. 如果参加同学聚会，大部分人都吸烟，你会一起吸吗？				
c. 当一个尊敬的人或长辈给你一支烟，你会吸吗？				

动机因素

18. 你是否因为好奇尝试吸烟？

A：是

B：否

19. 你是否因为周围人吸烟而尝试吸烟?

A：是

B：否

20. 你是否认为吸烟使人看起来更有吸引力?

A：是

B：否

21. 你是否认为吸烟可以带来愉悦?

A：是

B：否

22. 你是否认为吸烟可以缓解压力?

A：是

B：否

23. 你是否认为只要坚持锻炼，吸烟就不会危害健康?

A：是

B：否

24. 你是否认为戒烟会不利于人际关系?

A：是

B：否

机会因素

25. 你是否与父母同住?

A：是

B：否

26. 你的父母吸烟吗?

A：两个都不吸

B：两个都吸

C：其中一方吸

D：不知道

27. 你的好朋友或比较亲近的同学中是否有人吸烟?

A：是

B：否

28. 如果周围人给你递烟，你会吸吗?

A：是

B：否

29. 你是否在电视、录像/视频或者电影中看到有人吸烟?

A：是

B：否

30. 你是否在电视、手机、网络、广告牌等看到过关于烟草的商业促销广告?

A：是

B：否

控烟态度和行为

31. 你是否知晓学校的控烟政策?

A：是

B：否

32. 你是否认为学校应该实施控烟政策？

A：是

B：否

C：无所谓

33. 你认为目前学校实施的控烟政策是否有效？

A：是

B：否

C：不了解

34. 学校对于学生在校园吸烟行为处理方式是什么？

A：给予处分

B：通报批评

C：口头警告

D：不做处理

E：其他（_____）

35. 学校实施控烟政策后，你是否吸烟？

A：周末回家吸

B：校内隐蔽场所吸

C：照常

D：不吸烟

36. 你是否愿意参加校内开展的控烟健康教育？

A：是

B：否

37. 你认为在公共场合吸烟是否正常?

A：是

B：否

38. 如果你是被动吸烟者，你会选择?

A：立即走开

B：不予理会

C：进行劝阻

39. 你对学校目前的控烟政策是否有建议或意见?

____

感谢你参加本次调查!

Questionnaire on Adolescents' Tobacco Control, Health Behaviors and Attitudes

Dear Students:

Hello! We are the research team for the Survey on Adolescents' Tobacco Control, Health Behaviors and Attitudes from the School of Public Health, Lanzhou University. This survey is intended to investigate adolescents' health behaviors and attitudes towards tobacco control, so as to provide a basis for the improvement and implementation of campus tobacco control policies.

This survey adopts anonymous response. There are no right or wrong answers to your responses. The collected data will only be used for this research, and personal information will not be disclosed through any channels. If you agree to participate, please tick the box below and click "Next" to start the questionnaire.

The Research Team for the Survey on Adolescents' Tobacco Control, Health Behaviors and Attitudes

July 2025

Thank you for your participation! Please read carefully and answer the following questions. Unless otherwise specified, most questions are single-choice—please select the option that best fits your situation. For open-ended questions, please answer in the provided text box.

Basic Information

1. What is your grade?

A: Grade 2023

B: Grade 2024

2. What is your major?

A: Engineering Cost

B: Food Inspection and Testing Technology

C: Intelligent Construction Technology

D: Construction Project Management

E: Grain Engineering Technology and Management

F: Grain Storage, Transportation and Quality Safety

G: Construction Economy Informatization Management

3. What is your gender?

A: Male

B: Female

4. How old are you? _____ years old

5. What is your household registration location?

A: Urban area

B: Rural area

6. What is your approximate monthly living expenses? _____ RMB (excluding tuition fees)

7. How do you perceive your health status?

A: Good

B: Fairly good

C: Average

D: Fairly poor

E: Poor

Tobacco Use Status

8. Did you smoke before the start of this semester?

A: Yes

B: No

9. (Skip logic: Only answer if you selected "Yes" for Question 8) Approximately how many cigarettes did you smoke per day previously? _____

10. Fagerstrom Test for Nicotine Dependence (FTND) (Below are some descriptions related to daily life. Please select the option that best matches your recent situation based on facts and mark "√" in the corresponding box.)

Assessment Content	0 Points	1 Points	2 Points	3 Points
a. How long after waking up in the morning do you smoke your first cigarette?	After 60 minutes	Within 31-60 minutes	Within 6-30 minutes	Within 5 minutes
b. Do you find it difficult to control the urge to smoke in non-smoking areas?	No	Yes		
c. Which cigarette are you most unwilling to give up?	Others	The first one in the morning		
d. How many cigarettes do you smoke per day?	10 or fewer	11-20	21-30	31 or more
e. Do you still smoke when you are sick?	No	Yes		
f. Do you smoke more in the first hour after waking up than at other times?	No	Yes		

11. Where do you usually smoke?

A: At home

B: In the school dormitory

C: On the school playground

D: In the teaching building

E: Outdoor areas on campus

F: In the school toilet

G: Other places (please specify: _____)

Ability Factors

12. Which of the following health problems do you think smoking may cause?* (Multiple choices allowed)

A: Stroke

B: Osteoporosis

C: Lung cancer

D: Heart attack

E: Yellowing of teeth

F: Decreased sperm quality in males

G: Brain development damage

H: Amblyopia

13. Which of the following health problems do you think secondhand smoke inhalation (passive smoking, i.e., the involuntary inhalation of smoke exhaled by other smokers) may cause?* (Multiple choices allowed)

A: Lung cancer

B: Respiratory tract infection

C: Stroke

D: Heart disease

E: Bronchitis

F: Asthma

G: Otitis media

14. Do you know about e-cigarettes?

A: Yes

B: No

15. Have you ever smoked e-cigarettes?

A: Yes

B: No

16. Do you think e-cigarettes are harmful to the human body?

A: Yes

B: No

17. Self-Efficacy Assessment (Below are some descriptions related to self-perception of smoking. Please select the option that best matches your recent situation based on facts and mark "√" in the corresponding box.)

Assessment Content	Definitely not |	Probably not	Probably yes	Definitely yes
a. If a good friend offers you a cigarette, will you smoke it?				
b. If you attend a classmate gathering where most people are smoking, will you join them?				
c. If a respected person or elder offers you a cigarette, will you smoke it?				

Motivational Factors

18. Did you try smoking out of curiosity?

A: Yes

B: No

19. Did you try smoking because people around you smoke?

A: Yes

B: No

20. Do you think smoking makes people look more attractive?

A: Yes

B: No

21. Do you think smoking can bring pleasure?

A: Yes

B: No

22. Do you think smoking can relieve stress?

A: Yes

B: No

23. Do you think that as long as you keep exercising, smoking will not harm your health?

A: Yes

B: No

24. Do you think quitting smoking will be detrimental to interpersonal relationships?

A: Yes

B: No

Opportunity Factors

25. Do you live with your parents?

A: Yes

B: No

26. Do your parents smoke?

A: Neither of them smokes

B: Both of them smoke

C: One of them smokes

D: I don't know

27. Do any of your good friends or close classmates smoke?

A: Yes

B: No

28. If someone around you offers you a cigarette, will you smoke it?

A: Yes

B: No

29. Have you seen people smoking on TV, videos, or movies?

A: Yes

B: No

30. Have you seen commercial promotion advertisements for tobacco on TV, mobile phones, the internet, billboards, etc.?

A: Yes

B: No

Attitudes and Behaviors Towards Tobacco Control

31. Are you aware of the school's tobacco control policy?

A: Yes

B: No

32. Do you think the school should implement a tobacco control policy?

A: Yes

B: No

C: It doesn't matter

33. Do you think the current tobacco control policy implemented by the school is effective?

A: Yes

B: No

C: I don't know

34. What is the school's handling method for students smoking on campus?

A: Give disciplinary action

B: Circulate a criticism

C: Verbal warning

D: Take no action

E: Others (_____)

35. Do you smoke after the school implemented the tobacco control policy?

A: Smoke when going home on weekends

B: Smoke in concealed areas on campus

C: Smoke as usual

D: Do not smoke

36. Are you willing to participate in tobacco control health education carried out on campus?

A: Yes

B: No

37. Do you think smoking in public places is normal?

A: Yes

B: No

38. If you are a passive smoker, what will you choose to do?

A: Leave immediately

B: Pay no attention

C: Persuade them to stop

39. Do you have any suggestions or opinions on the school's current tobacco control policy?

____

Thank you for participating in this survey!
